# Mechanical Performance of Knitted Hollow Composites from Recycled Cotton and Glass Fibers for Packaging Applications

**DOI:** 10.3390/polym13142381

**Published:** 2021-07-20

**Authors:** Hafsa Jamshaid, Rajesh Mishra, Muhammad Zeeshan, Bilal Zahid, Sikandar Abbas Basra, Martin Tichy, Miroslav Muller

**Affiliations:** 1 Faculty of Textile Engineering, National Textile University, Faisalabad 37610, Pakistan; hafsa@ntu.edu.pk (H.J.); z.muhammad25@yahoo.com (M.Z.); basra.ntu@gmail.com (S.A.B.); 2 Department of Material Science and Manufacturing Technology, Faculty of Engineering, Czech University of Life Sciences Prague, Kamycka 129, 6-Suchdol, 165-00 Prague, Czech Republic; martintichy@tf.czu.cz (M.T.); muller@tf.czu.cz (M.M.); 3 Textile Engineering Department, NED University of Engineering and Technology, Karachi 75270, Pakistan; drbilalzahid@neduet.edu.pk

**Keywords:** recycled cotton, glass, hollow knitted composite, compression, flexural modulus, impact energy

## Abstract

This research deals with the development of knitted hollow composites from recycled cotton fibers (RCF) and glass fibers (GF). These knitted hollow composites can be used for packaging of heavy weight products and components in aircrafts, marine crafts, automobiles, civil infrastructure, etc. They can also be used in medical prosthesis or in sports equipment. Glass fiber-based hollow composites can be used as an alternative to steel or wooden construction materials for interior applications. Developed composite samples were subjected to hardness, compression, flexural, and impact testing. Recycled cotton fiber, which is a waste material from industrial processes, was chosen as an ecofriendly alternative to cardboard-based packaging material. The desired mechanical performance of knitted hollow composites was achieved by changing the tube diameter and/or thickness. Glass fiber-reinforced knitted hollow composites were compared with RC fiber composites. They exhibited substantially higher compression strength as compared to cotton fiber-reinforced composites based on the fiber tensile strength. However, RC fiber-reinforced hollow composites showed higher compression modulus as compared to glass fiber-based composites due to much lower deformation during compression loading. Compression strength of both RCF- and GF-reinforced hollow composites decreases with increasing tube diameter. The RCF-based hollow composites were further compared with double-layered cardboard packaging material of similar thickness. It was observed that cotton-fiber-reinforced composites show higher compression strength, as well as compression modulus, as compared to the cardboard material of similar thickness. No brittle failure was observed during the flexural test, and samples with smaller tube diameter exhibited higher stiffness. The flexural properties of glass fiber-reinforced composites were compared with RCF composites. It was observed that GF composites exhibit superior flexural properties as compared to the cotton fiber-based samples. Flexural strength of RC fiber-reinforced hollow composites was also compared to that of cardboard packaging material. The composites from recycled cotton fibers showed substantially higher flexural stiffness as compared to double-layered cardboard material. Impact energy absorption was measured for GF and RCF composites, as well as cardboard material. All GF-reinforced composites exhibited higher absorption of impact energy as compared to RCF-based samples. Significant increase in absorption of impact energy was achieved by the specimens with higher tube thickness in the case of both types of reinforcing fibers. By comparing the impact performance of cotton fiber-based composites with cardboard packaging material, it was observed that the RC fiber-based hollow composites absorb much higher impact energy as compared to the cardboard-based packaging material. The current paper summarizes a comparative analysis of mechanical performance in the case of glass fiber-reinforced hollow composites vis-à-vis recycled cotton fiber-reinforced hollow composites. The use of recycled fibers is a positive step in the direction of ecofriendly materials and waste utilization. Their performance is compared with commercial packaging material for a possible replacement and reducing burden on the environment.

## 1. Introduction

Textile structural composites are becoming more and more dominant as alternative materials to replace conventional load bearing materials, e.g., metal, wood, or concrete, due to their high performance to weight ratio. These materials are multifunctional in nature due to outstanding mechanical and physical properties which can be specially designed and engineered to meet the specific performance requirements of a particular application area. Textile-reinforced composite materials can exhibit excellent resistance to corrosion, wear, and even resistance to degradation at high temperatures [1]. These modern materials have a very wide range of applications in modern life, including sports equipment, automobiles, aeronautical components, buildings, infrastructure, etc. A composite material consists of two or more constituent materials which are mixed or bonded on a macroscopic level while the interfaces are in microscopic scale. Normally, a load bearing composite consists of a reinforcement which can be fibers, particles, flakes, or fillers. The reinforcement is impregnated in a matrix that can be polymers, metals, or ceramics, depending on the application area. When manufactured properly, the new combined material exhibits properties superior to the constituent materials [2].

Reinforcement used for composites can be natural, as well as synthetic, fibrous materials. The advantage of synthetic fiber-reinforced composite is its high strength and mechanical properties, which are more suitable to use in structural applications. The main problems associated with synthetic fiber-reinforced composites are the environmental aspects, e.g., production process, application, and afterlife disposal. It is harmful to the environment because it is not biodegradable and is made from nonrenewable resources [3]. Due to environmental concerns, a large amount of research is directed towards natural fibers. The prime reason for selection of natural fibers in new product development is their minimal contribution to the greenhouse effect. The most important concern is to protect our environment from pollution, and it can be achieved without compromising the performance and quality of the product. The solution is to use biodegradable materials which are obtained from natural and renewable sources. Due to environmental concerns, plant fiber-reinforced composites are receiving greater attention of researchers and industrialists because they are biodegradable, combustible, and lightweight [4]. Recently, natural fiber-reinforced composites have received great attention from researchers and industrialists as a replacement of synthetic fiber-reinforced composite. They have relatively good mechanical and physical characteristics that can be used in various applications. Natural fibers are bio-degradable, nonabrasive, nonhazardous, lightweight, and renewable materials [5]. Researchers are focusing on development of products from recyclable materials due to increasing environmental concern. In this context, recycled natural fiber-based textile waste can be used as a sustainable material in composite reinforcement. Several researchers have successfully used recycled cotton/polyester material to produce composites with significant mechanical and acoustic performance level [6,7]. Value added composite samples were also developed by using recycled cotton fiber waste from discarded denim fabrics. These materials exhibit sufficient thermal, acoustic, and mechanical performance [8,9].

Researchers have prepared thermoplastic composites using natural flax and thermoplastic polypropylene yarn on flat bed knitting machine by developing plain and rib structures and compared their mechanical properties. Three-dimensional knitted fabric reinforcement was developed on flat knitting machine for thermoplastic composites using glass and PP filaments. Results showed that mechanical properties of composites are affected by knitting structure and direction of inlay yarn [10,11].

Textile-reinforced hollow composites have numerous applications, such as sports equipment, pipes, drive shafts, printing rollers, landing gears for helicopters, rocket structure, structural building components, etc. Hollow composite tubes can be prepared by several methods, e.g., knitting, spacer weaving, braiding, stitching, etc. Researchers have investigated the deformation and fracture behavior of glass-epoxy braided circular tubes for different loading cases, like compression, torsion, combined tension-torsion, compression-torsion, etc., both experimentally and theoretically [12,13]. Other researchers proposed models for simulating the crushing behavior and predicting the energy absorption characteristics of triaxially braided carbon fiber/epoxy-vinyl ester composite tubes with both circular, as well as square, cross-sections [14]. Braided hollow textile preforms were used for development of composites by several researchers [15,16]. Many others have investigated the hybridization of glass woven fabric with a natural fiber mat for applications in the piping industry for commercial applications. Researchers have investigated the flexural stiffness of thick composite tubes which were manufactured by using an automated fiber placement machine [17].

Several researchers used finite element modeling for analysis of bending strength of cylindrical composites. They developed an enhanced version of finite element models (FEM) for elastic and non-linear plastic analysis of tubes [18]. In literature, models for both straight and curved composite tubes have been presented [19]. A beam element for analysis of straight and tapered composite tubes under general loading was investigated [20]. A model for four-point bending of a thermoplastic composite using a three-dimensional solid element was reported [21]. The lateral planar crushing and bending responses of carbon fiber-reinforced plastic (CFRP) square tube filled with aluminum honeycomb was investigated. The square tube was developed from plain weave of carbon fiber. Good agreement was obtained between numerically predicted results by FEM and experimentally measured results [22].

Knitting is well established as a technique to produce hollow and tubular structures. However, there is relatively very small amount of research conducted to use such structures for development of hollow/tubular composites. Despite the easy shaping capability of knitted fabrics, published literature is generally focused on the reinforcement of composites with knitted plates or flat structures. An advantage of knitted fabric-based hollow composites is the possibility of multiple layering by intermeshing of the loop structure in-between the different fabric layers. The structural maneuverability and compressional resilience in knitted hollow composites offers several research opportunities and product development possibilities. Such a research gap in the literature provided motivation for experimental investigation of hollow composites produced by knitting technology.

There is limited understanding of the mechanical properties and long-term durability of hollow knitted composites for application as structural components. In the current research, the mechanical properties of glass (GF)-reinforced hollow composites are investigated by varying the tube diameters for use in secondary structural elements, such as wall panels or door systems. Its advantages, such as being light weight, non-corrosive, and low maintenance cost, make such composites suitable alternative for steel, wood, and concrete materials.

A further objective of the present study is to develop knitted hollow composite preforms by using recycled cotton (RC) yarn, which has not been reported in literature. These composites are aimed at applications relating to packaging of heavy components. For packaging of heavy/bulkier goods, cardboards are normally used because of their cushioning properties. Cardboard is developed from paper which needs wood as a raw material; thus, it is not an environmentally-friendly option. A typical packaging is made of three layers of heavy paper, two flat layers with a wavy/corrugated one in the middle. Although it is hard enough not to break or tear, it cannot be reused. Moreover, it is not easily recyclable and dumped as landfill. Even the decomposition of cardboard materials generates methane, which is a major greenhouse gas with a global warming capacity 21 times more powerful than carbon dioxide. Its recycling also contributes to environmental pollution by using different sources of energy. During recycling, it needs almost 75% of the energy needed to make new one, while the quality is much inferior. In view of the existing issues, hollow knitting is a promising technology to prepare composite preforms which can replace conventional packaging materials based on hard paper. Use of recycled cotton obtained from industrial waste is a sustainable approach towards minimizing environment pollution.

Different mechanical tests, e.g., hardness, compression, flexural, and impact measurement, were carried out for the developed composite samples. The mechanical properties of knitted hollow composites developed from glass fiber were compared with hollow composites developed from recycled cotton fibers. Further, the cotton fiber-based samples were compared with cardboard packaging material of similar thickness. The findings provide new opportunities for an ecofriendly alternative material with superior cushioning, as well as protective performance.

## 2. Materials and Methods

### 2.1. Materials

**Figure 1:**
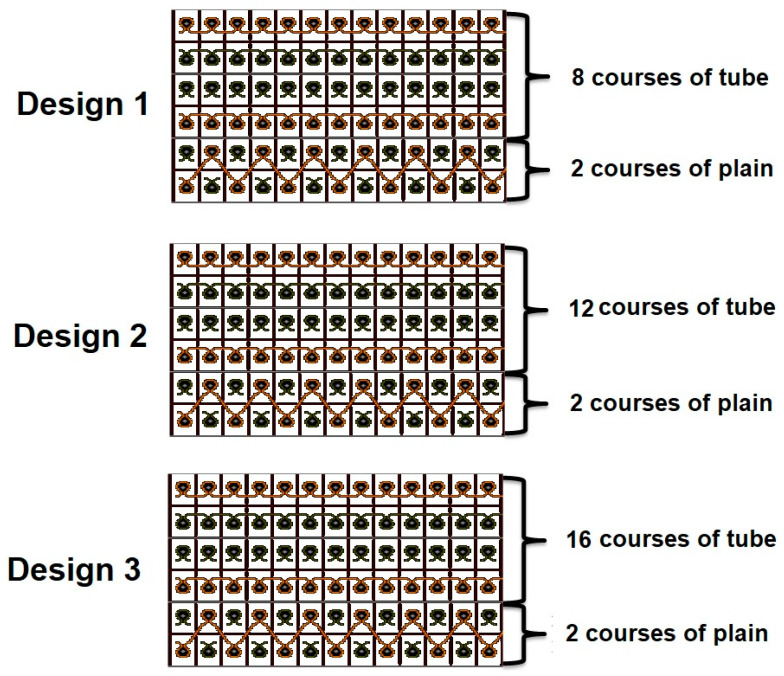
Knitting design (short representation).

Knitted hollow fabrics were developed by using recycled cotton (RC) fiber and glass (G) fiber yarn of 1800 denier on a V-bed flat knitting machine using gauge 7E. During formation of plain courses, both front and back needle beds remain operational simultaneously. However, during tube formation, both needle beds knit separately, as shown in Figure 1.

All knitted tubular sample designs were developed on the SDS-ONE APEX platform [23]. Tubes with different thickness were developed by using different numbers of courses (8, 12, and 16). In total, six types of knitted preforms were developed/manufactured. Details of the fabric manufacture are given in Table 1. These specifications were selected in order to achieve the composite sample thickness similar to the thickness of commercially available double-layered carboard packaging material.

### 2.2. Methods 

#### 2.2.1. Production of Composite Samples

**Figure 2:**
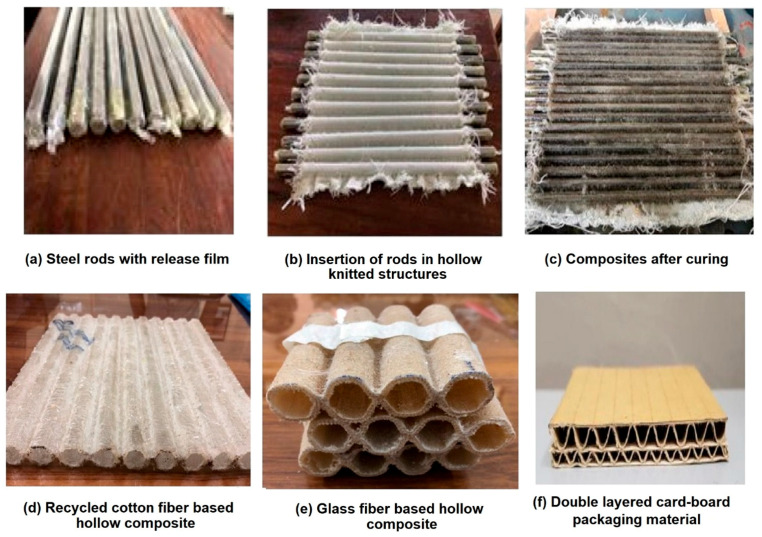
Photographs of developed composite samples versus double-layered cardboard.

Unsaturated polyester resin (UPR) (KZN Resins, Durban, South Africa), Malikens GP 555-04, which is dissolved in a 35 + 2% of organic liquid solvent “styrene”, was used for preparation of composite samples. This is a thermoset polymer which, when oxidized, starts to convert from liquid to a gel, and later, to a hard solid form. Cobalt octoate (KZN Resins, Durban, South Africa), which is a metal salt of carboxylic acid, was used as an accelerator in the curing process of the polyester resin. MEKP 50 (Methyl ethyl ketone peroxide) (KZN Resins, Durban, South Africa), which is an organic substance, was used as hardener in the process. Composite samples were developed by inserting hexagonal metal pipes into the hollow fabrics. The metal pipes were first covered with plastic release film, as shown in Figure 2a, for easy removal of sample after composite manufacturing. After successful insertion of metal rods into the hollow fabric samples, as shown in Figure 2b, impregnation with resin was carried out. The impregnated samples were dried for 24 h and cured for 4 h at 100 °C. After the curing operation, the inserted pipes were removed, and the composite samples were obtained, as shown in Figure 2c. The internal diameters of the manufactured hollow composite samples were 8 ± 0.02 mm, 10 ± 0.02 mm, and 14 ± 0.02 mm, respectively, for both glass fiber- and recycled cotton fiber-based samples. Outer diameter was equivalent to that of double-layered cardboard sample. The test samples were cut from fabricated composites according to different standard requirements. The images of RC fiber knitted hollow composite, glass fiber knitted hollow composite, and conventional cardboard packaging material are shown in Figure 2d–f, respectively.

Mechanical behavior of commercially available double-layered cardboard (W) packaging material was also investigated in order to compare with recycled cotton fiber-based sample (C2), which is of most similar thickness (outer diameter).

**Figure 3:**
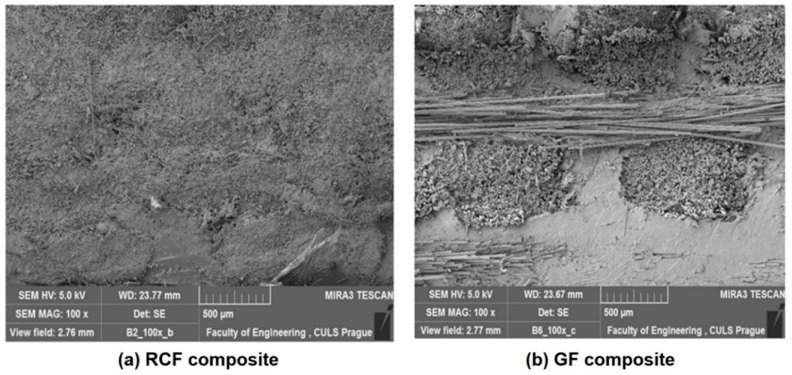
SEM images of cross-sections: (**a**) RCF sample, (**b**) glass fiber sample.

Scanning electron microscopy (SEM) (NIST, Gaithersburg, MD, USA) images of recycled cotton fiber (RCF)- and glass fiber (GF)-based composite sample cross-sections are shown in Figure 3a,b, respectively.

These images indicate that the samples are free from any major voids, and the fibers are properly impregnated with the resin. The uniformity of impregnation also ensures a strong interface between the fibrous phase and the matrix phase.

#### 2.2.2. Characterization

**Figure 4:**
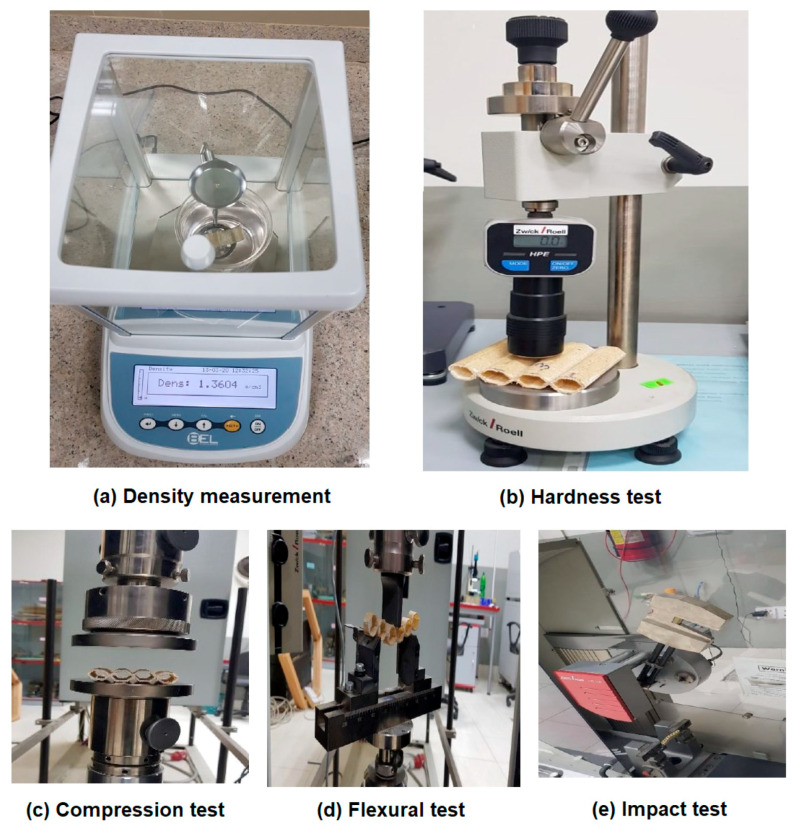
Physical and mechanical characterization of manufactured hollow composite samples.

The density of the natural fiber-based composite materials using a polymeric matrix can be determined according to the standard ASTM D 792, using an analytical balance equipped with a stationary support for the immersion vessel, as shown in Figure 4a. In this method, a solvent, such as water or propanol, can be used as immersion liquid, depending on the density of the polymer.

In this investigation, the density was measured by using water as the immersion liquid. The samples absorb some water through the micro pores. In fact, the textile structures and their composites are almost always composed from micro pores, which can absorb water. Initially, the dry mass of the composite samples was measured. Then, the hollow composite sample was immersed in the water column of known volume. The overall increase in water level (or volume) indicates the volume occupied by the solid (non-porous) portion of the composite. That means the sample occupies a space equivalent to its solid volume (not including its porosity). About 1 h of time was allowed for complete immersion and penetration of water through all possible pores. Then, the final water level was noted. The sample was taken out of the water column. Further, the mass of wet composite sample was measured, which is slightly higher than the dry mass of the same sample. The difference of mass indicates the mass of water absorbed through the micro pores. The volume corresponding to this mass difference is calculated and added to the solid volume of the composite sample in order to obtain the overall volume of the composite. It should, however, be noted that the water absorption capacity of developed composite samples was relatively much smaller as compared to their primary knitted structures and was only found to be around 1–2%. Density of composite samples was calculated from the dry mass and the overall volume, including micro pores. All the results of measurements are presented in Table 2.

Specimens of suitable dimensions were cut as per specified standards for physical and mechanical testing. Fiber volume fraction (Vf) of recycled cotton fiber-based hollow composite was maintained to be approximately 40%, and, for glass fiber-based composites, it was approximately 50%. The surface hardness of all the samples developed was measured by Barcol Hardness tester of Zwick/Roel, Brno, Czech Republic, as shown in Figure 4b, according to standard ASTM D2583. The test specimen is placed under the indenter of hardness tester, and uniform pressure of 1 bar was applied as per standard. Twenty measurements were conducted, and the mean value was calculated.

Mechanical characterization, e.g., compression, flexural test, and impact measurements, were carried out for all the developed hollow composite samples as they are the most essential performance requirements for packing applications.

**Figure 5:**
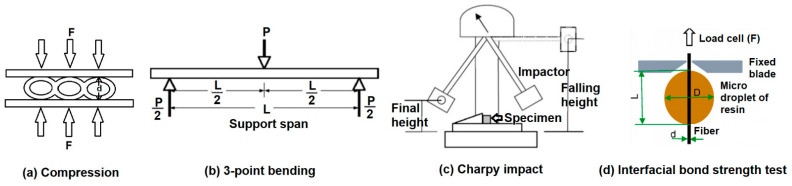
Principles of (**a**) compression test, (**b**) 3-point bending test, (**c**) Charpy impact test, and (**d**) fiber-matrix interfacial bond strength test.

Compression strength indicates the resistance of a material to deformation under pressure. The compression strength of all the hollow composite specimens, as well as cardboard material, was determined by using Universal Testing Machine (Z100-100 KN) manufactured by Zwick/Roell, Brno, Czech Republic, as shown in Figure 4c, according to the standard ASTM D2412-11, at a crosshead speed of 1.3 mm per minute. This test determines the compression load-deflection characteristics of hollow composite samples subjected to loading between two parallel steel plates, as shown in Figure 5a. The measurements were repeated 20 times, and the mean value was calculated. The compression strength and strain were obtained using Equations (1) and (2), respectively. It was assumed that the tube will become elliptical during the load application [24].

(1) $$\mathrm{Compression}\;\mathrm{strength}=\frac{F}{\Delta_{y}}{\left(1+\frac{\Delta_{y}}{2d}\right)}^{3},$$

(2) $$\mathrm{Compressive}\;\mathrm{strain}=\frac{\Delta_{y}}{d}\times 100\%,$$

where: F is the applied load, d is the outside diameter, and $\Delta_{y}$ is the change in the outside diameter of the specimen in the load direction. Equation (3) was used to calculate the compression modulus.

(3) $$\mathrm{Compression}\;\mathrm{modulus}=0.149\;r^{3}\times \mathrm{Compression}\;\mathrm{strength}.$$

The flexural behavior of the composite samples was evaluated by using the 3-point bending test by using Universal Testing Machine (Z100-100 KN), by Zwick/Roell, Brno, Czech Republic as shown in Figure 4d, according to the test method of ASTM D-7264. The same method was also used for evaluation of the double-layered cardboard packaging material. A specimen of rectangular shape having dimensions 120 mm × 13 mm was supported at the ends and deflected at the center point. As force was applied on the specimen, and it started deflecting from the center, its deflection and force were measured and recorded until the failure occurred or the maximum force reduced to 40%. The principle of 3-point bending is shown in Figure 5b. The gauge length/support span of 80 mm, deformation rate of 1 mm/min, and load of 5 kN was maintained. Twenty measurements were conducted, and the mean value was calculated. The flexural strength was calculated using Equation (4) [25].

(4) $$\sigma =3\mathrm{PL}/2{\mathrm{bh}}^{2}.$$

Flexural modulus was calculated using Equation (5)

(5) $$E={\mathrm{PL}}^{3}/4{\mathrm{ybh}}^{3},$$

where P represents Load, L represents gauge length, b represents width, h represents thickness, and y represents deflection or strain during bending.

The Charpy impact test was performed by following the ISO-179 standard testing procedure. An impact testing machine (Model HIT50P) manufactured by Zwick/Roell, Germany, as shown in Figure 4e, was used for the test. A swinging hammer/pendulum with 21 J energy and velocity of 3.8 m/s was used to test the specimens for impact energy. The hollow composite specimens, as well as cardboard material, were tested without a notch. Samples were cut into size 80 mm × 10 mm for testing. The thickness and width of the samples were measured by Vernier caliper before the test. Specimens were placed on the specific slot, and the pendulum was allowed to impact in order to hit and break the specimen, as shown in Figure 5c. The measurements were repeated 20 times, and the mean value was calculated [26]. Impact energy was calculated as:

E = Mass of impactor (m) × acceleration due to gravity (g) × falling height (h),(6)

Impact energy absorbed =

Energy of striking impactor − Residual energy of rebounding impactor.(7)

In order to determine the fiber-matrix interfacial bond strength, the single fiber pull out using the microdroplet test was conducted [2,27]. The single fibers of recycled cotton, as well as glass, were treated with microdroplets of the resin. Then, the fibers were cured under similar conditions as the composite samples. The impregnated fiber samples were dried for 24 h and cured for 4 h at 100 °C. The diameter of the microdroplets of resin was around 50 μm. The principle of microdroplet test to determine interfacial bond strength is shown in Figure 5d. The interfacial bond strength was calculated using Equation (8).

(8) Interfacial bond strength=F/π d L,

where F is the maximum load, d is the average fiber diameter, and L is the length of fiber embedded in the droplet of resin. Ten measurements were carried out for cotton, as well as glass fibers, and the mean was calculated. The interfacial bond strength for cotton fiber was found to be 28.52 ± 0.2 MPa, and that for glass fiber was 19.35 ± 0.2 MPa. The stronger interface of cotton fiber with the unsaturated polyester resin can be attributed to the relatively rough fiber surface as compared to a smoother surface of the glass fiber.

## 3. Results and Discussion

### 3.1. Surface Hardness

It was observed that the surface hardness of glass fiber-based hollow composites and recycled cotton fiber-based samples are almost similar. In fact, the surface is mostly composed of the resin, and the fibers are embedded deeper inside. Therefore, the hardness of the surface is mainly dominated by the cured-resin hardness. All the hollow composites exhibit almost 7–8 times higher hardness as compared to double-layered cardboard material. This is an indication of longer service life in case the of the knitted hollow composites as compared to paper-based conventional packaging material. Further, it was observed that the hardness slightly decreases as the diameter of the tube increases. It can be attributed to decreasing curvature, which reduces the stiffness. It is well known that rigidity is inversely proportional to the radius of curvature. However, it must be noted that the change of surface hardness in this case is only marginal.

### 3.2. Compression Properties

The compression strength is one of the major properties required in composites used in packaging applications. It is generally accepted that fiber strength is the most important parameter responsible for composite strength. During mechanical testing, fiber fracture happens when the force exceeds the limiting strength of the fiber and interfacial bonding with the resin.

**Figure 6:**
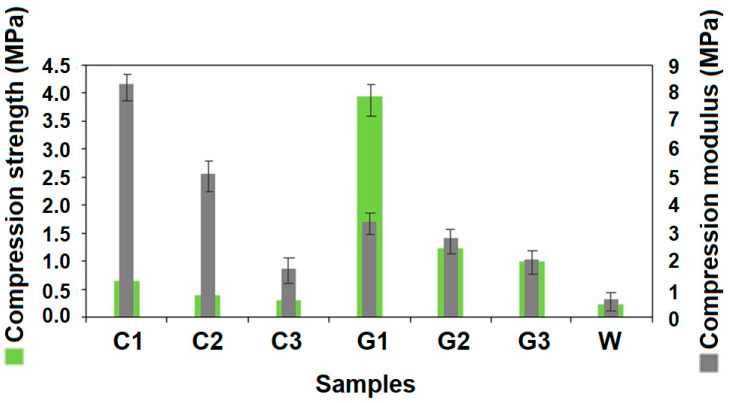
Compression strength and modulus of RC and glass fiber-reinforced hollow composites versus double-layered cardboard.

As glass is a relatively stronger fiber, its composites also exhibit substantially higher compression strength as compared to RC fiber composites. The trend is clearly visible from Figure 6.

It is well known that glass fibers are more crystalline and more rigid as compared to natural origin cellulosic fibers as cotton. Therefore, a glass fiber-based composite offers higher stiffness and compressional strength as compared to recycled cotton fiber-based composites. It should be noted that the fineness/linear density of both glass and cotton yarns are the same. Moreover, the GF-based composite samples are developed with higher fiber volume fraction (Vf = 50%) as compared to RCF-based samples (40%). Thus, the fiber mechanical properties and the fiber volume fraction have significant influence on the overall composite mechanical performance. Such observations are also validated by the rule of mixture and the Halpin-Tsai equations shown below [28].

(9) $$K_{c}=K_{m}\left[\frac{1+\xi \zeta V_{f}}{1-\eta V_{f}}\right],$$

(10) $$\mathrm{With},\;\eta =\left[\frac{(K_{f}/K_{m})-1}{(K_{f}/K_{m})+\zeta }\right],$$

where *Kc* represents the effective compressional (mechanical) property of the composite, while *Kf* and *Km* are the corresponding fiber and matrix compressional (mechanical) properties, *Vf* denotes the fiber volume fraction, and ζ is a geometrical parameter, which represents the reinforcement geometry, packing geometry, and loading conditions. In the present analysis, the geometry is defined by the knitting pattern, and yarn fineness, which is same for both types of materials.

Compression strength of both RC- and glass fiber-reinforced composites show an inverse trend with increasing tube diameter. This fact is governed by basic relations in bending/compressional deformation. During compression, the hollow segment undergoes ovalization, and the tubes undergo bending deformation. Bending rigidity is always higher for a lower radius of curvature. Therefore, the smaller tube diameter results in higher stiffness, as well as compression strength. Sample C1, having the lowest diameter of 11.8 mm, shows 60.2% and 20.9% higher compression strength as compared to C2 and C3, respectively. Similarly, G1 show 966% and 292% higher compression strength as compared to G2 and G3, respectively. During the compression test, buckling is the main phenomenon responsible for failure of fiber reinforced hollow composites [29]. As the composite structure undergoes compression, the assembled fibers and yarns tend to spread and become misaligned. Higher diameter of the tube reduces curvature of the fibers and yarns on the surface. Thus, they are susceptible to deform to a higher extent during compression. The samples of hollow composites having higher diameter tend to offer more severe buckling phenomena and relatively lower resistance to compression load. The outcome is lower compression strength. These observations are also supported by previously reported literature [25,26]. All the developed hollow composite samples exhibit higher compression strength as compared to double-layered cardboard packaging material.

It is interesting to note that RC fiber-reinforced hollow composites show higher compression modulus as compared to glass fiber-reinforced composites. This observation is in contrast with the findings about compression strength. As is well known, modulus is a derived parameter which depends on both compressive stress and compressive strain. The shorter fiber length in recycled cotton enables much lower deformation compared to relatively much longer glass fibers during the compression test. In addition, it must be noted here that the tubes made from RC-based materials have a wall thickness almost twice that of glass fiber-based composite tubes. Higher thickness in this case is also obvious due to the higher thickness of cotton yarns, pertaining to lower density as compared to glass. Therefore, even with much lower compression strength, RC fiber-reinforced hollow composites exhibit significantly higher compression modulus. The knitted prepregs of RC also prove to be strongly bonded with the resin as per results of microdroplet test. Further, in the SEM image presented in Figure 3, a more uniform and deeper impregnation is observed in RCF-based samples as compared to GF-based samples. The hollow composite sample (C2) exhibits higher compression strength and significantly higher compression modulus as compared to the double-layered cardboard (W) packaging material of similar thickness. Thus, they can be easily used as replacement of cardboard-based packaging material with much superior compressional properties.

**Figure 7:**
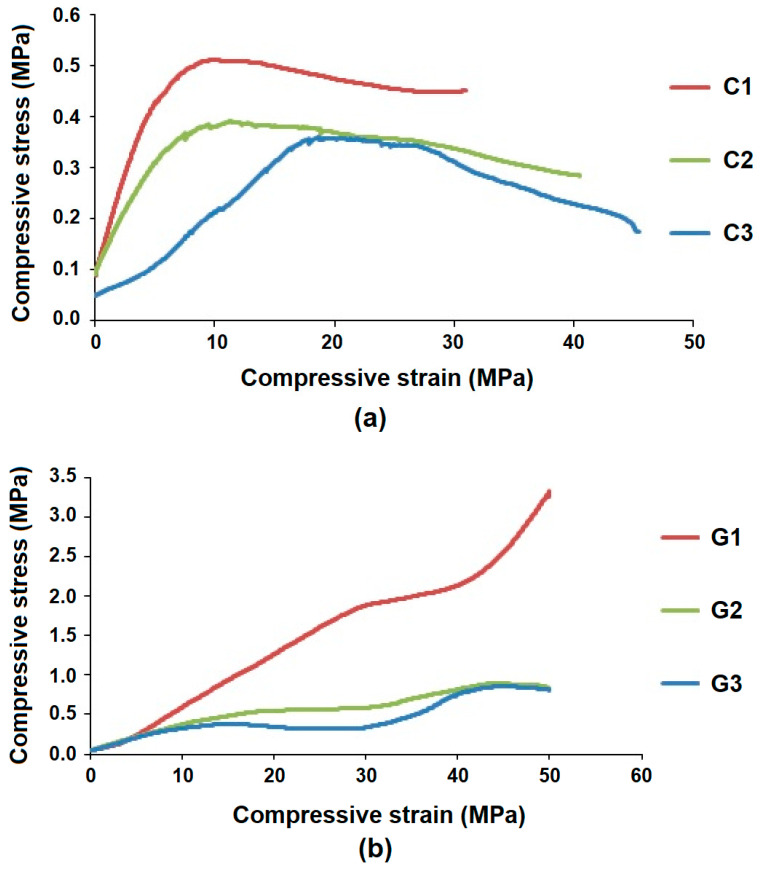
Compressive stress versus strain for (**a**) recycled cotton fiber-based hollow composites and (**b**) glass fiber-based hollow composites.

The differences in the stress-strain behavior during compression test for RC fiber composites and glass fiber composites are shown graphically in Figure 7a,b, respectively.

The compression stress-strain curves for the glass fiber-based hollow composites indicate much higher compression strength or peak compressive stress level. The inherent mechanical properties of glass, which are undoubtedly much higher than the recycled cotton fiber, are responsible for such behavior. It is also visible that the curves for RCF-based samples show higher slope values as compared to GF-based samples. This is indicative of the higher compression modulus in RCF-based hollow composites. This behavior is attributed to lower level of deformation before peak compression load. Shorter fiber length in recycled cotton and deeper impregnation of resin are the factors responsible for higher compression modulus observed in RCF-based hollow composites. Sample C1 offers higher stress compensation as compared to C2 and C3. It exhibits a permanent deformation after 8% compression, as shown in Figure 7a. Sample C1 shows the highest maximum compressive stress and lowest compressive strain due to the smallest tube diameter among the recycled cotton fiber-based samples. Smaller tube diameter, along with shorter RC fibers, enables the structure to bear higher compressive stress. This is due to the fact that both the upper and lower arms are connected by a shorter fiber column, which provides higher resistance to the applied compression load. Further the short fibers on the upper and lower surface can effectively align themselves and absorb the stresses. In the case of higher tube diameter, and longer fibers, there is more flattening and a higher chance of fiber slippage. These observations are also validated by reported literature [24,25,27,28].

Similarly, among the glass fiber-reinforced composites, G1 shows the highest strength and peak stress level due to its smallest tube diameter, as shown in Figure 7b. The compressional strength increases with lower radius of curvature. The performance is governed by the geometry, which is defined by curvature, tube diameter, wall thickness, etc., as defined in the Halpin-Tsai equations [28].

Furthermore, all the glass fiber-based hollow composites exhibit two peaks in their compression curve, as shown in Figure 7b. The stress-strain behavior concerning the decrease and increase of the stiffness in glass fiber-reinforced hollow knitted composites are not completely unexpected. There are several research studies reported in literature where the compression behavior of knitted structures and their composites are described [30,31,32,33,34,35,36]. The initial part of the compression curve denotes the elastic stage, which corresponds to flattening and ovalization of hollow channels. The middle part showing a slight decrease is known as the plateau stage, which corresponds to deformation at the joining points. The third part, which again shows an increasing trend, denotes to the densification of fibers and load transfer to matrix. The dual peaks are more distinct in the sample (G3), which has the maximum tube diameter and thickness among all GF-based hollow composites. The first peak, which is observed at around 15% compressive strain, corresponds to the maximum elastic limit of the tubular structure. During this phase, the reinforcing fibers tend to spread and absorb the compressive stress. The first peak corresponds to the jamming state, which is the maximum limit before load transfer to the matrix phase. Subsequently, there is stress concentration at the weakest links in the hollow composite. These points are located at the joints and contact area between adjacent tubes. Stress concentration at these points results in deformation at the joining points of upper and lower half of the tubes, which results in a second peak at approximately 40% deformation level. These double peaks during compression test can be justified by the shape change in the tubes. The shape change (ovalization) effect is visible by flattening of the circular shape of the tube. This, in turn, decreases the stiffness of the composite structures. Such observations are also supported by reported literature [30,31,32,33,34,35,36]. In samples G1 and G2, the peaks are not as distinct as in G3 due to lower tube thickness/diameter. There is less flattening in samples of lower diameter of the hollow tubes. They offer more resistance to compressional deformation by virtue of the stiffness resulting from lower radius of curvature. Since the cotton fibers are relatively weaker, they fail/break before the second phase of compression occurs. Thus, RCF-based samples show only one distinct peak in the compression curve. Among the three RCF-based hollow composites, only sample C3 shows a slightly visible second peak. This is attributed to maximum diameter and flattening of the hollow tubes, which enables absorption of a small compressive stress in the second phase, though the overall peak stress is the minimum.

**Figure 8:**
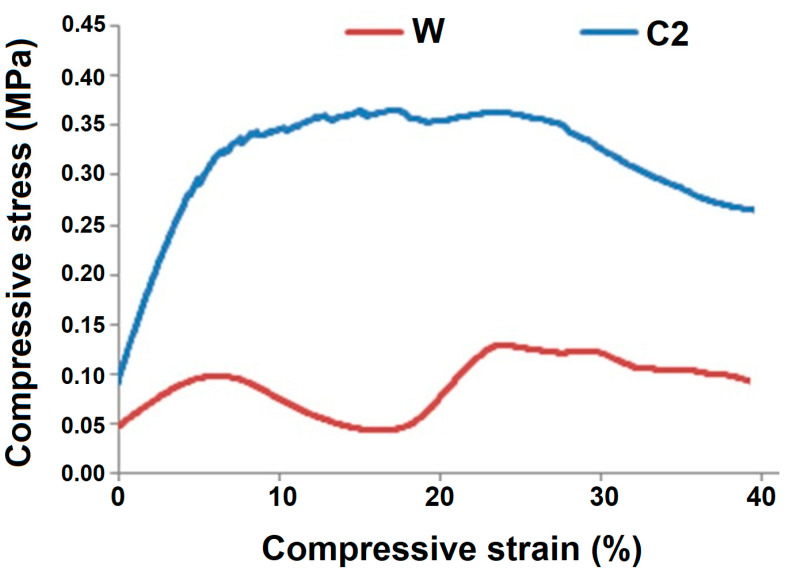
Compression behavior of RC fiber composite (C2) and cardboard material (W).

The compression behavior of the commercially available double-layered cardboard-based sample W was also tested and compared with the RCF-reinforced hollow composite sample (C2) having the nearest thickness. The comparison of the stress-strain curves is shown in Figure 8.

The sample C2 performs three times better than paperboard under compression load. This can be attributed to the strong interface between recycled cotton fibers and the polymer resin, as observed from SEM image in Figure 3. Further, the interfacial bond strength between recycled cotton fiber and the resin has also been measured by microdroplet test and found to be significantly high. As a result, the hollow columns of cotton fiber-based composites provide much better protection against relatively larger compressive stresses. The curved tubular hollow channels in the developed composite samples offer higher level of resistance to compressional deformation. Moreover, the reinforcing fibers can absorb the compressive stresses more effectively. On the other hand, the cardboard-based material is weaker and offers minimal resistance. Based on the rule of mixture and the Halpin-Tsai models, it can be predicted that fiber-reinforced composites can offer higher mechanical performance as compared to the constituent elements [25,26,27,28]. The cardboard material is relatively weaker, and there is absence of strong inter-polymer linkage as in fibers and polymeric resins.

The dual peak behavior is visible in double-layered cardboard as in the case of GF-based samples. The first peak is result of flattening and stress absorption. After the flattening of the cardboard paper material, the stress is accumulated at the joints of the cells/hollow tubes. The paper walls tend to buckle and bend. This buckling action enables further absorption of compressive stress. Thus, the second peak of stress is slightly higher than the first peak. Overall, paper-based hollow packaging material proves to be weaker and less resistant to compressive deformation.

### 3.3. Flexural Properties

**Figure 9:**
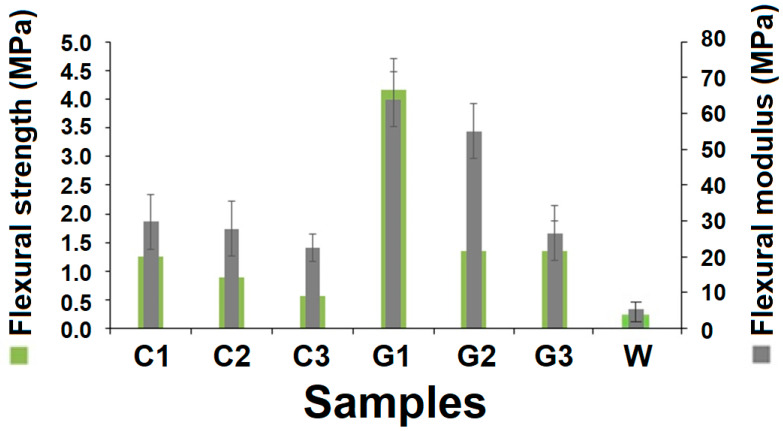
Flexural strength and modulus of RC and GF-reinforced hollow composites versus double-layered cardboard material.

The failure of composites under flexural loading involves a combination of tensile failure, compression failure, shear, and/or delamination at different levels [25,26]. When a sample is subjected to bending deformation, the outer surface experiences tensile stress, while the inner surface experiences longitudinal compression. In the case of multiple layers of fibers, the tensile stress propagates inwards and causes delamination, which ultimately reduces the flexural resistance/strength. A comparative account of flexural behavior for all RCF- and GF-reinforced hollow composite samples, along with double-layered cardboard material, is shown in Figure 9.

Flexural strength also follows a similar decreasing trend as that of compression strength with the increase of tube diameter. The bending deformation translates into partial compression; thus, the trend is similar. Under 3-point bending mode, the support span undergoes a deflection as the load is applied. Initially, the load is taken up by the reinforcing fibers, which experience an extension on the outer layer and inward compression on the inner layer. Thus, the macroscale bending behavior is a cumulative response of the fiber tensile/elastic modulus and moment of inertia. Such behavior is reported by several other researchers in the available literature [24,25,26,27].

The flexural strength of GF-reinforced samples is much higher as compared to RCF-based samples. This is traced back to stronger glass fibers and relatively higher fiber volume fraction in glass fiber-reinforced hollow composites in the current study. The rule of mixture and the Halpin-Tsai model are validated for bending performance, as well [28].

From Figure 9, it is clearly visible that the flexural strength of C1 (1.24 MPa) is higher than C2 (0.89 MPa) and C3 (0.53 MPa). Similarly, flexural strength of G1 (4.16 MPa) is higher than that of G2 (1.36 MPa) and G3 (1.35 MPa), respectively. The smaller tube diameter enables the sample to withstand higher level of bending stress. According to fundamentals of bending, the bending rigidity is inversely proportional to the fourth power of the tube. Thus, the higher curvature or lower radius of curvature is more beneficial to attain higher stiffness. Further, the glass fiber-reinforced hollow composites are substantially stiffer as compared to the recycled cotton fiber-based samples. Based on the Halpin-Tsai model, a higher bending rigidity of fibers and higher fiber volume fraction results in higher overall stiffness in the composite [26,27,28]. The glass fibers are much stiffer as compared to cotton fibers due to higher level of crystallinity. Thus, the bending performance of the constituent fibers is reflected in the flexural performance of the knitted hollow composites.

**Figure 10:**
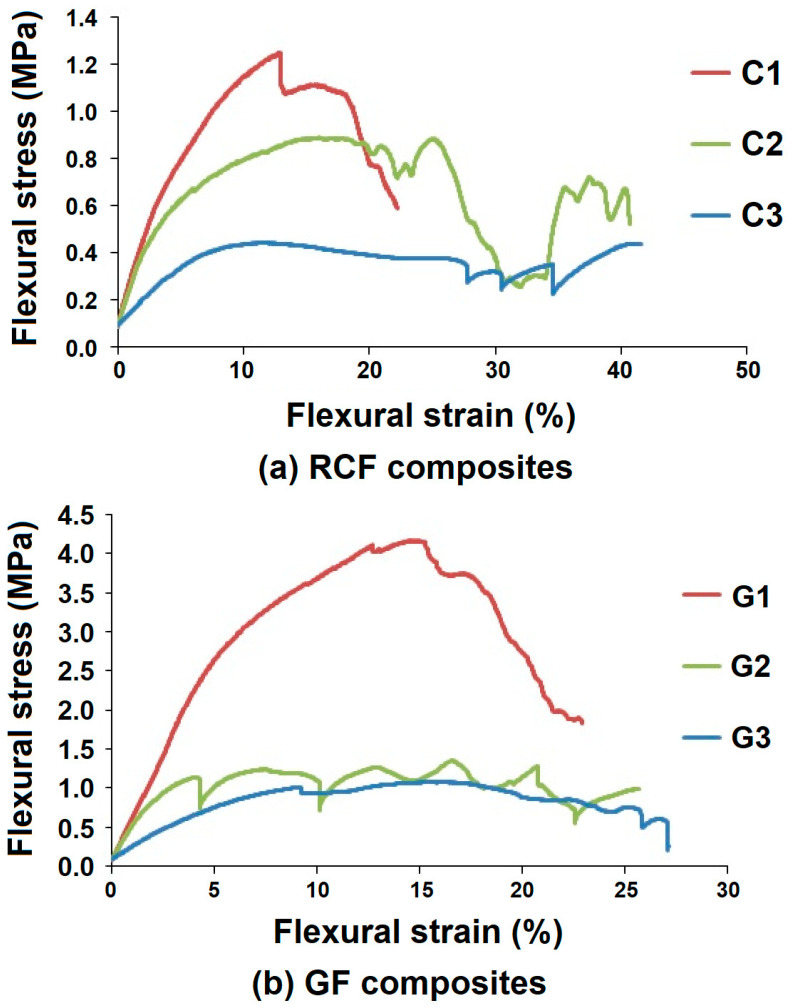
Flexural stress versus strain of (**a**) recycled cotton fiber-based hollow composites and (**b**) glass fiber-based hollow composites.

The flexural stress-strain behavior of RCF-based samples and GF-based samples are shown in Figure 10a,b, respectively. The figure shows that hollow composite samples C1 and G1 have relatively higher and steeper stress-strain curves as compared to other samples in their respective groups because of relatively smaller tube diameters. This is due to the fact that both the upper and lower arms are connected by a shorter continuous fiber column, which provides higher resistance to the applied bending load. The weak link in tubular channels are the inter-tube joints. With smaller diameters, the link is smaller and does not allow any fracture to initiate. Further, the fibers on the upper and lower surface can effectively align themselves and absorb the stresses. In the case of higher tube diameter, there is more flattening and a higher chance of fiber slippage. These observations are also validated by reported literature [24,25,27,28].

The maximum bending stress is absorbed by samples C1 and G1 with a deformation of 13% and 15%, respectively. On the other hand, C2, C3 and G2, G3 manage maximum stresses at a deformation of 24%, 40% and 16%, 17%, respectively, as shown in Figure 10. It can be observed that higher tube diameter enables higher flexural strain. This is indicative of the fiber slippage and flattening in samples C2, C3 and G2, G3. Further, the stress-strain curves show multiple steps in the case of higher diameters. Such behavior is observed for both RCF- and GF-based samples. This can be attributed to a step wise stick-slip behavior exhibited by the fibers in the hollow composites. These peaks also indicate matrix cracking, cracking of the supporting layer, and ovalization effect as discussed previously in the compression study. Such observations are also reported by other researchers [24,26,27].

The slope of the flexural curves defines the flexural modulus, which also indicates stiffness. It can be observed that samples C1 and G2 show maximum stiffness in their respective groups of samples. Such observations are supported by theoretical models, as well as experimental studies, reported in the literature [28]. During the flexural test, no brittle failure was observed (especially in specimens with higher tube diameter). This is indicative of very good service performance of such hollow composites as a packaging material.

**Figure 11:**
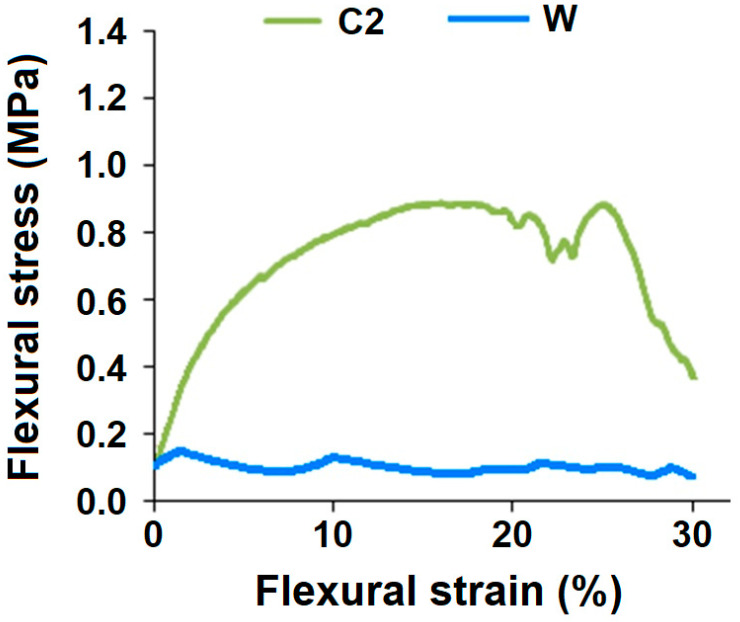
Flexural behavior of RC fiber-based hollow composite (C2) and cardboard material (W).

Since sample C2 has the nearest thickness as that of the cardboard-based material, their flexural performance is compared in Figure 11.

The flexural stress-strain curves of RC fiber-reinforced hollow composite sample (C2) and double-layered cardboard material (W) is shown in Figure 11. The cardboard material exhibits a rather flat stress-strain behavior. The material cannot survive higher flexural stresses. On the other hand, a fiber structure-based hollow composite shows substantially higher flexural rigidity, even with the use of recycled cotton material. Thus, it is deemed to be suitable to replace the cardboard in the packaging applications. Textile structural composites show geometry related performance and can be designed in special way to perform load bearing functions. The results are validated by theoretical models reported in the literature [13,21,28].

### 3.4. Impact Properties

**Figure 12:**
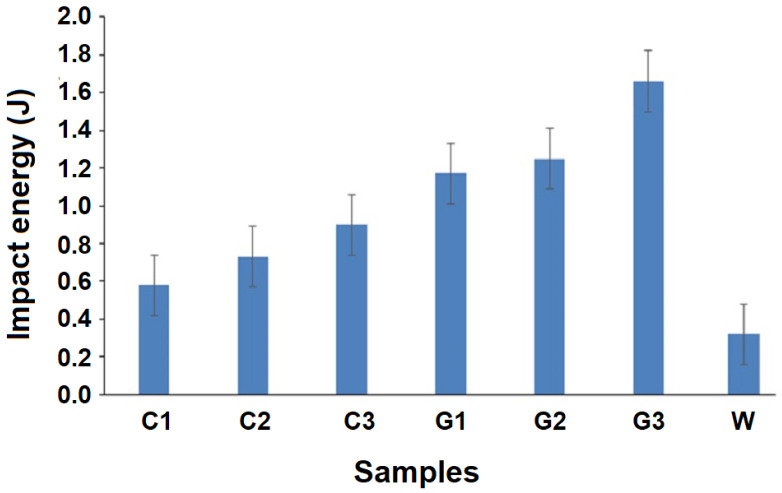
Impact energy absorbed by RC and glass fiber-based hollow composites versus double-layered cardboard.

Impact testing helps to understand the primary cause of failure or delamination of composites due to sudden impact of stone pellets, metal edges, rods, baggage loading, and dropping, or even during maintenance. Fiber mechanical properties have a very significant influence on impact properties of the textile-reinforced composites since these are the primary load bearing elements. These reinforcing elements absorb major portion of the energy during impact. The impact properties of the composite materials are directly related to their toughness [37,38,39,40]. Unnotched specimens of GF-reinforced hollow composites were not at all broken during the Charpy test in the present investigation. A comparative account of impact energy absorbed by the hollow composite specimens developed from RC fiber and glass fiber, as well as double-layered cardboard, is given in Figure 12.

Figure 12 indicates a significant increase in impact energy absorbed by the specimens with increase in tube diameter/thickness. This behavior is observed in the case of RCF-based, as well as GF-based, hollow composites. Such improvement in impact energy absorption with increasing tube diameter is in striking contrast to the results obtained in compression and bending tests. In general, impact energy absorbed by a sample depends upon its ability to deform/extend over a longer period of time and, thus, to absorb the impact energy for the total work done during this deformation. Among the recycled cotton fiber-based samples, impact energy absorption for C3 is 23.3% higher than C2 and 55.2% higher than C1, respectively. Similarly, among the glass fiber-reinforced hollow composites, impact energy absorbed increases with an increase in tube diameter. Sample G3 shows 25.3% higher impact energy absorption as compared to sample G2, and 43.1% higher absorption than G1. Such behavior can be explained in terms of the flattening and ovalization of hollow composites having higher diameter of the tubes. By such structural deformations, the hollow composites with higher thickness/diameter of tubes are able to absorb higher amount of energy exerted by the impactor. On the other hand, samples with smaller tube diameter proved to be stiffer and less capable of absorbing impact energy. The observations of compressional and flexural performance support such behavior. A sample which is relatively more compressible (lower resistance to compression) and relatively less stiff proves to be more efficient absorber of impact energy. The maximum absorption of the impact energies by samples C3 and G3 in their respective groups is because of higher tube diameters, which offer higher deformation/elongation before attaining peak stress. The higher impact energy is also attributed to the change in momentum of the hammer. Samples C3 and G3 having higher tube diameter provide longer impact time before peak force, hence absorbing the highest amount of energy. The behavior can be traced back to the hardness results given in Table 2. In order to absorb higher impact energy, the material needs to be softer and allow higher deformation under impact. However, the hardness is a surface property and depicts resistance to localized surface deformation only. A softer surface initiates the energy absorption, which is further enhanced with higher tube thickness/diameter. On comparing the results of the hardness tests with impact tests, a negative trend is obtained. Higher surface hardness is associated with lower absorption of impact energy. Similar results are also reported in the literature [27]. However, further deeper analysis will be needed in this aspect.

In general, glass fiber-based hollow composites exhibit higher impact energy absorption as compared to the recycled cotton fiber-reinforced composite samples. This is mainly due to higher tensile strength and modulus of glass fibers, as well as the higher fiber volume fraction with respect to the recycled cotton fiber-based hollow composites. The Halpin-Tsai model based on the rule of mixture can be successfully used to predict such performance in fiber-reinforced composites [28].

All the knitted hollow composites developed exhibit substantially higher absorption of impact energy as compared to the cardboard-based packaging material. The cardboard-based packaging material is composed of weaker cellulosic material, which has relatively inferior mechanical performance. On the other hand, fiber-reinforced composite structures, and more specifically knitted hollow composites, are very efficient cushioning materials, which exhibit an efficient absorption mechanism [41,42]. It can be noted that the cardboard materials completely broke down and were destroyed after the impact test. Therefore, such conventional packaging materials cannot be reused, whereas the hollow composite samples did not break completely. While recycled cotton fiber-reinforced samples developed minor cracks in some cases, glass fiber-based samples showed absolutely no sign of any structural damage. They can probably be used in even higher risk packaging purposes as aircraft components, military equipment, automotive parts, sports equipment, etc. The use of recycled cotton fibers is a sustainable and ecofriendly approach, while reducing the environmental burden of cardboard-paper-based packaging materials.

**Figure 13:**
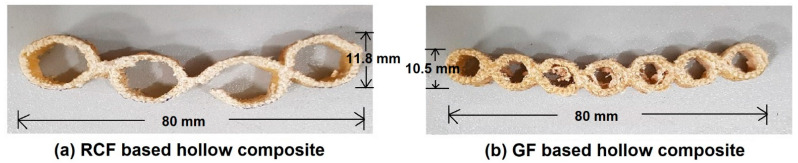
Ovalization after impact testing in (**a**) recycled cotton fiber-based hollow composites and (**b**) glass fiber-based hollow composites.

The ovalization effect after the impact test in the case of RC fiber- and glass fiber-reinforced hollow composite is shown in Figure 13.

Though the RC fiber-based hollow composites shown in Figure 13a develop some cracking at a few joints, the glass fiber-reinforced composite sample in Figure 13b is completely undamaged.

## 4. Conclusions

In the current study, an attempt has been made to develop knitted hollow composites by using recycled cotton fibers and glass fibers. Composite samples developed were subjected to hardness test, compression, flexural, and impact loading. Glass fiber-reinforced hollow composites exhibit substantially higher compression strength as compared to RC fiber-based composites. However, RC fiber-reinforced hollow composites show higher compression modulus as compared to glass fiber-based samples due to shorter fiber length, which enables much lower deformation during compression loading.

Compression strength of both RC- and glass fiber-reinforced composites decreases with increasing tube diameter. The RCF-based hollow composites were compared with a commercial cardboard-based packaging material of equivalent thickness. Substantially higher compression strength, as well as compression modulus, was observed in RCF-based hollow composites as compared to the double-layered cardboard packaging material of similar thickness. No brittle failure was observed during the flexural test, and samples with smaller tube diameter exhibited higher stiffness. RC fiber-reinforced hollow composites show substantially higher flexural stiffness as compared to double-layered cardboard material. Significant increase in absorption of impact energy was achieved by the specimens with higher tube diameter. The ovalization and flattening effect enable higher absorption of impact energy. The RC fiber-based hollow composites absorb much higher impact energy as compared to the cardboard-based packaging material. The findings are in accordance with previous research and theoretical models based on the rule of mixture, as well as the Halpin-Tsai model.

RC fiber-based hollow composite is proven as a replacement for paperboard packaging material in order to utilize industrial waste and reduce the environmental pollution. Theses composites can be used as separation/packaging material for heavy goods. Recycled cotton is more appropriate and ecofriendly alternative for cardboard-based packaging. In addition, such composites can be used repeatedly as their performance and durability are much higher than cardboard packaging materials.

Glass fiber-based hollow composites can be used in relatively higher load bearing applications, e.g., separator for offices, kiosks, boats, and light weight shelters. Its sound insulation properties can also be improved by inserting porous sound absorbing material inside the tubes. These tubes can be filled with foam or any honeycomb structures for construction elements in body parts of lightweight electric vehicles, which can survive crash/impact to reasonably a higher extent. In composite structures, the honeycomb core plays a vital role in energy absorption properties. In the case of hollow structures, the fabric consists of one or more layers of triangular, trapezoidal, or hexagonal cross-sectional shapes, which are self-opening. These geometrical variations will further enhance the applicability of hollow composites in several engineering applications. This research opens new directions for further investigation on hollow composites with different core geometries and core fillings.

## Figures and Tables

**Table 1:** Knitted fabric parameters.

Sample ID	Yarn Type	Linear Density of Yarn (Denier)	Plain Courses	Tube Courses	Wales (cm^−1^)	Courses (cm^−1^)	Stitch Length (cm)
C1	Recycled Cotton (RC)	1800	2	8	11	18	0.71
C2	Recycled Cotton (RC)	1800	2	12	11	18	0.71
C3	Recycled Cotton (RC)	1800	2	16	11	18	0.71
G1	Glass (G)	1800	2	8	12	16	0.69
G2	Glass (G)	1800	2	12	12	16	0.69
G3	Glass (G)	1800	2	16	12	16	0.70

**Table 2:** Physical parameters of the developed composite samples.

Sample ID	Fiber Volume Fraction (Vf %)	Density (g/cm^3^)	Hardness (Barcol) Scale of (0–100)	Diameter of Tube	
				Inner Dia (mm)	Outer Dia (mm)
C1	40 ± 2	1.23 ± 0.02	82.7 ± 0.2	8.1 ± 0.1	11.8 ± 0.1
C2	40 ± 2	1.21 ± 0.02	81.9 ± 0.2	10.2 ± 0.1	14.8 ± 0.1
C3	40 ± 2	1.21 ± 0.02	80.1 ± 0.2	14.3 ± 0.1	18.5 ± 0.1
G1	50 ± 2	1.41 ± 0.02	88.0 ± 0.2	8.2 ± 0.1	10.5 ± 0.1
G2	50 ± 2	1.42 ± 0.02	87.7 ± 0.2	10.2 ± 0.1	12.5 ± 0.1
G3	50 ± 2	1.39 ± 0.02	86.6 ± 0.2	14.2 ± 0.1	16.5 ± 0.1
W	-	-	11.5 ± 0.2	-	15.0 ± 0.1

## Data Availability

Not applicable.
